# BNIP3-Dependent Mitophagy via PGC1α Promotes Cartilage Degradation

**DOI:** 10.3390/cells10071839

**Published:** 2021-07-20

**Authors:** Deokha Kim, Jinsoo Song, Eun-Jung Jin

**Affiliations:** 1 Department of Biological Sciences, College of Natural Sciences, Wonkwang University, Iksan 54538, Jeonbuk, Korea; ejrgk29@naver.com (D.K.); songjinsoo85@naver.com (J.S.); 2 Integrated Omics Institute, Wonkwang University, Iksan 54538, Jeonbuk, Korea

**Keywords:** PGC1A, miR-126-5p, BNIP3, autophagy, mitophagy, osteoarthritis

## Abstract

Since mitochondria are suggested to be important regulators in maintaining cartilage homeostasis, turnover of mitochondria through mitochondrial biogenesis and mitochondrial degradation may play an important role in the pathogenesis of osteoarthritis (OA). Here, we found that mitochondrial dysfunction is closely associated with OA pathogenesis and identified the peroxisome proliferator-activated receptor-gamma co-activator 1-alpha (PGC1α) as a potent regulator. The expression level of PGC1α was significantly decreased under OA conditions, and knockdown of PGC1α dramatically elevated the cartilage degradation by upregulating cartilage degrading enzymes and apoptotic cell death. Interestingly, the knockdown of PGC1α activated the parkin RBR E3 ubiquitin protein ligase (PRKN)-independent selective mitochondria autophagy (mitophagy) pathway through the upregulation of BCL2 and adenovirus E1B 19-kDa-interacting protein 3 (BNIP3). The overexpression of BNIP3 stimulated mitophagy and cartilage degradation by upregulating cartilage-degrading enzymes and chondrocyte death. We identified microRNA (miR)-126-5p as an upstream regulator for PGC1α and confirmed the direct binding between miR-126-5p and 3′ untranslated region (UTR) of PGC1α. An in vivo OA mouse model induced by the destabilization of medial meniscus (DMM) surgery, and the delivery of antago-miR-126 via intra-articular injection significantly decreased cartilage degradation. In sum, the loss of PGC1α in chondrocytes due to upregulation of miR-126-5p during OA pathogenesis resulted in the activation of PRKN-independent mitophagy through the upregulation of BNIP3 and stimulated cartilage degradation and apoptotic death of chondrocytes. Therefore, the regulation of PGC1α:BNIP3 mitophagy axis could be of therapeutic benefit to cartilage-degrading diseases.

## 1. Introduction

Osteoarthritis (OA) is the most common chronic joint disease caused by articular cartilage loss, subchondral sclerosis, and abnormalities of the synovial membrane and periarticular structures [1]. The pathogenesis of OA is characterized by extracellular matrix (ECM) degradation and cellular stress that lead to the activation of proinflammatory cytokines [2,3] or dysfunction of cellular organelles such as endoplasmic reticulum (ER) [4,5], peroxisome [6,7], and mitochondria [8,9,10]. Among the various cellular organelles, mitochondria are one of the most important organelles in eukaryotic cells. Mitochondria regulate important cellular function and cell survival that may have a key role in age-related diseases [11]. Since articular chondrocytes are highly glycolytic cells, the role and function of mitochondria have not been well-studied until recently. Recent studies suggested that the dysfunction and degradation of mitochondria could be associated with OA [12,13,14,15]. In OA chondrocytes, decreased activity of mitochondrial respiratory complex and mitochondrial mass was observed [16,17]. Mitochondrial biogenesis is also deregulated in OA chondrocytes [15,18,19].

Autophagy may play a crucial role in maintaining cartilage homeostasis and the deregulation of autophagy could contribute to cartilage degradation, a hallmark of OA pathogenesis [20]. Autophagy is a cellular self-protection mechanism by removing damaged organelles and proteins and is known to be regulated by a series of autophagy-related genes, such as BECN1 and microtubule-associated protein 1 light chain 3 (LC3) [21].

Several reports suggested that autophagy is stimulated in OA chondrocytes [22]. However, our laboratory and others have reported that autophagy is significantly suppressed in OA chondrocytes and this suppressed autophagy is linked to the cell death of chondrocytes [6,23,24]. One organelle-specific autophagy is mitophagy, the selective degradation of mitochondria through the macroautophagy pathway [25,26]. In general, mitophagy can be divided into Parkin RBR E3 ubiquitin protein ligase (PRKN)-dependent and PRKN-independent pathways [27]. To date, most studies have focused on PINK1/PRKN-mediated mitophagy [28,29,30,31,32,33]. PRKN-dependent mitophagy is mediated by the PTEN induced putative kinase 1 (PINK1) and the E3-ubiquitin ligase PRKN [28]. Normally, translocated PINK1 into the inner mitochondrial membrane is cleaved by presenilin-associated rhomboid-like protein and N-terminal truncated PINK1 is degraded. However, the loss of mitochondrial transmembrane potential accumulates uncleaved PINK1 by disrupting the translocation of PINK1 and this accumulated PINK1 recruits and activates the PRKN that amplifies mitophagy signaling in the cytoplasm [28,29].

It is known that PINK1-mediated mitophagy is provoked in various biological and pathological conditions [30,31,32]. PINK1-mediated mitophagy is closely associated with cell death in human primary chondrocytes and *Pink1* knockout mice showed significantly reduced cartilage degeneration in response to the intra-articular injection of monosodium iodoacetate (MIA) to induce OA [33]. On the other hand, PRKN-independent mitophagy is primarily depending on receptor proteins that interact with LC3 and GABARAP through an LC3-interacting region (LIR) motif of the BCL2 interacting protein 3 (BNIP3) and BCL2/adenovirus E1B interacting protein 3-like (BNIP3L/NIX) [34,35]. It has been suggested that the accumulation of reactive oxygen species (ROS) activates BNIP3L/NIX-mediated mitophagy [36]. Moreover, in cardiac progenitor cells (CPCs), PRKN-dependent mitophagy did not affect the programmed mitophagy during differentiation. Rather, PRKN-independent mitophagy through BNIP3L and FUNDC1 is involved in CPCs [37]. BNIP3L is known to play a critical role in removing mitochondria during erythroid maturation [38,39] and in differentiating retinal ganglion cells [40]. In addition, little is understood about transcriptional regulation and potent regulatory factors of PRKN-independent mitophagy. It has been known that microRNA (miR)-137 inhibits mitophagy by targeting BNIP3L and FUNDC1 [41].

However, in the OA pathogenesis, the role and regulatory factor of PRKN-independent mitophagy have not been well studied. In this study, for the first time, we found the activation of PRKN-independent mitophagy due to upregulated peroxisome proliferator-activated receptor (PPAR)-γ coactivator α (PGC-1α) through the upregulation of BNIP3 during OA pathogenesis and identified that the miR-126-5p known OA-related microRNA (miR) is responsible for the upregulation of PGC-1α in the OA condition.

## 2. Materials and Methods

### 2.1. Animals and Experimental Osteoarthritis (OA)

Mice were maintained in a temperature and humidity-controlled room with 12 h/12 h day/night cycle. Osteoarthritic (OA) mouse cartilage was induced in eight-week-old mice by the destabilization of the medial meniscus (DMM) surgery using C57BL/6N mice. At 8–10 weeks after DMM surgery, cartilage tissues were processed for histological analysis.

### 2.2. Human Cartilage and Chondrocyte Culture

Human cartilage tissues were obtained from patients undergoing total knee replacement (TKR) and classified as relatively healthy (non-OA) or severely damaged (OA) regions. Non-OA and OA chondrocytes were extracted with collagenase and seeded into 10-cm culture dishes in Dulbecco’s Modified Eagle’s Medium (DMEM) supplemented with 10% FBS and 100 U/mL penicillin/streptomycin.

### 2.3. Lentivirus Packaging and Delivery

LentimiRa-Off-miR-126-5p vector was purchased from Applied Biological Materials, Inc. (ABM, Vancouver, BC, Canada; mh30100). Plasmid DNA was transfected into Lenti-X 293T cells (Clontech, Palo Alto, CA, USA; #632180) using Lentifectin (ABM, #G074) and a third-generation packaging mix (ABM, #LV053) in serum-free DMEM, and FBS was supplemented after 6–8 h. The supernatant contained with lentiviral particles was concentrated using a Lenti-X Concentrator (Clontech, #631232) and stored at −80 °C. For in vivo delivery, concentrated lentivirus (1 × 10^9^ PFU) was injected into the intra-articular joint cavity every week for eight weeks.

### 2.4. Histological Analysis

The patient and sacrificed mice cartilage samples were fixed with 10% neutral buffered formalin (NBF) for 24 h and decalcified using 0.5 M ethylenediaminetetraacetic acid (EDTA) solution for a week. After paraffin embedding, blocks were cut at 5-μm thickness and stained with safranin O. For immunohistochemical analysis, deparaffinized sections were incubated with primary antibodies overnight at 4 °C in a humidified chamber. Sections were subsequently developed using ImmPACT DAB (Vector Laboratories, Burlingame, CA, USA; #SK-4105). The following antibodies were used for immunohistochemical analysis; rabbit anti-Matrix Metallopeptidase 13 (MMP13) (Biovision, Milpitas, CA, USA; #3533, 1:200 dilution), rabbit anti-peroxisome proliferator-activated receptor gamma coactivator 1 (PGC1)α (Abcam, Cambridge, UK; #Ab54481, 1:200 dilution), rabbit anti- C1, 2C (IBEX Pharmaceuticals, Quebec, QC, Canada; #50-1035, 1:100 dilution), horseradish peroxidase (HRP)-conjugated goat anti-rabbit IgG (Enzo Life Sciences, Farmingdale, NY, USA; #ADI-SAB-300).

### 2.5. Immature Mice Articular Chondrocytes (iMACs) Culture

The primary culture using mouse articular cartilage was isolated from postnatal day 5 to 6 mice by dissection of the tibial plateaus and femoral condyles. Peeled cartilages were digested with 3 mg/mL of type IV collagenase solution (ThermoFisher Scientific, MA, USA; #17104019) for 45 min and transferred to a culture dish containing 0.5 mg/mL type IV collagenase solution and incubated overnight at 37 °C. Digested chondrocytes were filtered using a 70-μm cell strainer and cultured with low glucose DMEM medium supplemented with 10% FBS, 100 U/mL penicillin/streptomycin, and 2 mM L-glutamine at 37 °C in the presence of 5% CO_2_ for 6 days. Alcian blue staining was performed using 1% Alcian blue 8 GX in 0.1N HCl solution. Alcian blue was extracted with 6M guanidine-HCl, and quantified by measuring the absorbance of the extracts at 600 nm.

### 2.6. RNA Isolation and Quantitative Real-Time Polymerase Chain Reaction (qRT-PCR)

RNA was isolated using RNA isoplus (Takara, Mountain View, CA, USA; #9109) and reverse-transcribed using 5X All-in-One RT Master Mix (ABM, #G492). qRT-PCR was performed using AMPIGENE qPCR Green Mix (Enzo Life Sciences, #ENZ-NUC104-1000). The qRT-PCR primer sequences used in this study are listed in Supplementary Table S1 and *glyceraldehyde-3-phosphate dehydrogenase* (*Gapdh*) was used as an endogenous control. For miRNA detection, reverse transcription was performed using miScript II RT Kit (Qiagen, Venlo, The Netherlands; #218160) and qRT-PCR was performed using miScript SYBR Green PCR Kit (Qiagen, #218073) and Rnu6 was used as endogenous control. The expression levels were analyzed using StepOnePlus (ThermoFisher Scientific).

### 2.7. Western Blotting

Mitochondria or cytoplasmic proteins were performed using Mitochondria/Cytosol Fractionation Kit (Biovision, #K256-100) or RIPA lysis buffer (Cell Signaling, Beverly, MA, USA; #9806). The total protein concentration was measured using Bicinchoninic Acid (BCA) Protein Assay Kit (ThermoFisher Scientific, #23225). Protein lysates were separated by 10% or 12% SDS-PAGE and transferred to 0.2-μm nitrocellulose membrane (GE Health Care, Chicage, IL, USA; #10600004). The following antibodies were used for western blotting; rabbit anti-PGC1α (Abcam, #Ab54481, 1:1000 dilution), rabbit anti-autophagy related 12 (ATG12) (Cell Signaling, #4180, 1:1000 dilution), rabbit anti-Beclin 1 (Cell Signaling, #3495, 1:1000 dilution), rabbit anti-autophagy marker light chain 3 (LC3)B (Cell Signaling, #3868, 1:1000 dilution), rabbit anti-Parkinson’s disease 2 (PARK2) (MyBioSource, San Deigo, CA, USA; #MBS178284, 1:1000 dilution), rabbit anti-GAPDH (Bioworld Technology, St Louis Park, MN, USA; #AP0066, 1:5000 dilution), rabbit anti-translocase of outer mitochondrial membrane 20 (TOMM20) (Abcam, #ab186734, 1:1000 dilution), HRP-conjugated goat anti-rabbit IgG (Enzo Life Sciences, #ADI-SAB-300). The blots were visualized using a SuperSignal West Pico PLUS Chemiluminescent Substrate (ThermoFisher Scientific, #34579).

### 2.8. Monitoring of Autophagy and Mitophagy

For autophagy detection, iMACs were transfected with LC3-GFP plasmid vector at 5 days after chondrocyte seeding, and mitochondria were visualized using MitoTracker Red (ThermoFisher Scientific, #M22425) staining. For mitophagy detection, the pMitophagy Keima-Red mPark2 vector was purchased from MBL Life Science (#AM-V0259M) and performed according to the manufacturer’s protocols.

### 2.9. Annexin V & Dead Cell and MitoPotential Assay

The apoptotic cells were analyzed using Annexin V & Dead Cell Kit (Luminex, Austin, TX, USA; #MCH100105). Briefly, cells were collected, centrifuged at 300× *g* for 5 min, and resuspended in 100 μL of Annexin V and dead cell detection reagent (Merck Millipore, Billerica, MA, USA; #MCH100105) in phosphate-buffered saline containing 1% bovine serum albumin at room temperature for 20 min. The percentage of live, early apoptotic, late apoptotic, and dead cells were analyzed in accordance with the Millipore guidelines.

The mitochondrial depolarization state of treated cells was assessed using a Muse cell analyzer (Merck Millipore) using a MitoPotential assay kit (Luminex, #MCH100110). Briefly, both floating and adherent treated cells were collected, centrifuged at 300× *g* for 5 min, and then a 100 μL aliquot of cell suspension was first added to 95 μL of diluted Muse MitoPotential dye, and after 20 min at room temperature, 5 μL of 7-AAD reagent dye was added. After 5 min, the percentage of live, depolarized, and dead cells in the cell suspensions were measured immediately using a Muse cell analyzer.

### 2.10. Small Interfering RNA (siRNA) and microRNA (miRNA, miR) Transfection

The Pgc1a-specific siRNAs (siPgc1a-A, 5′-CUGACUUCGAGCUGUACUU-3′; siPgc1a-B, 5′-GAGUACUGAGAGUUGAGUA-3′; siPgc1a-C, 5′-GCACCAGAAAACAG CUCCA-3′) were purchased from Bioneer (Daejeon, Korea). The miR-126-5p mimic (5′-CATTATTACTTTTGGTACGCG-3′) and inhibitor (sense: 5′-CAUUAUUACUUUUGG UACGCG-3′; antisense: 5′-CGCGUACCAAAAGUAAUAAUG-3′) were purchased from Genolution (Seoul, Korea).

### 2.11. Luciferase Reporter Assay

The 3′UTR of Pgc1a was PCR amplified from genomic DNA of iMACs. The mutant type 3′UTR of *Pgc1a* oligo was purchased from Cosmogenetech (Seoul, Korea) and inserted into pMIR-Reporter vector (Ambion, Austion, TX, USA). For miR target validation, cells were transfected with each construct, miR-126-5p mimic or negative control (Scramble-miR).

## 3. Results

### 3.1. Upregulated PCG1a Is Responsible for OA Pathogenesis

The analysis of GSE16464 using Gene Set Enrichment Analysis (GSEA) showed a significant decrease in the expression level of mitochondrial genes in osteoarthritic (OA) chondrocytes compared to normal chondrocytes (Supplementary Figure S1A). Exposure of interleukin-1β (IL-1β) into immature murine articular chondrocytes (iMACs) to induced OA environment suppressed cartilage matrix synthesis as assessed by Alcian blue staining (Supplementary Figure S1B) as well the expression level of cartilage matrix genes such as *aggrecan* (*Acan*), *Collagen Type II Alpha 1 Chain* (*Col2a1*), and *cartilage oligomeric matrix protein* (*Comp*) (Supplementary Figure S1C) and stimulated the expression level of matrix degradation factor, such as *matrix metallopeptidase* (*Mmp*)*3*, *Mmp9*, and *Mmp13* (Supplementary Figure S1D) compared to control iMACs. We also observed deregulation of mitochondrial membrane potentials, i.e., increased level of depolarization and abnormal morphological alteration of mitochondria in the presence of interleukin (IL)-1β (Supplementary Figure S1E,F). Moreover, exposure of carbonyl cyanide 3-chlorophenylhydrazone (CCCP), uncoupler of mitochondrial oxidative phosphorylation that depolarizes the plasma membrane and reduces ATP production was also increased depolarization of the mitochondrial membrane and abnormal morphological alteration of mitochondria as seen in IL-1β treated iMACs (Supplementary Figure S2A,B). Cartilage matrix as assessed by Alcian blue staining (Supplementary Figure S2C) and the expression level of genes involved in the synthesis of the cartilage matrix such as *Acan*, *Col2a1*, and *Comp* were also significantly decreased with CCCP treatment (Supplementary Figure S2D). The treatment of IL-1β and CCCP increased the apoptotic death of chondrocytes (Supplementary Figure S2E). These data suggest that the deregulation of mitochondria function is associated with OA pathogenesis.

**Figure 1:**
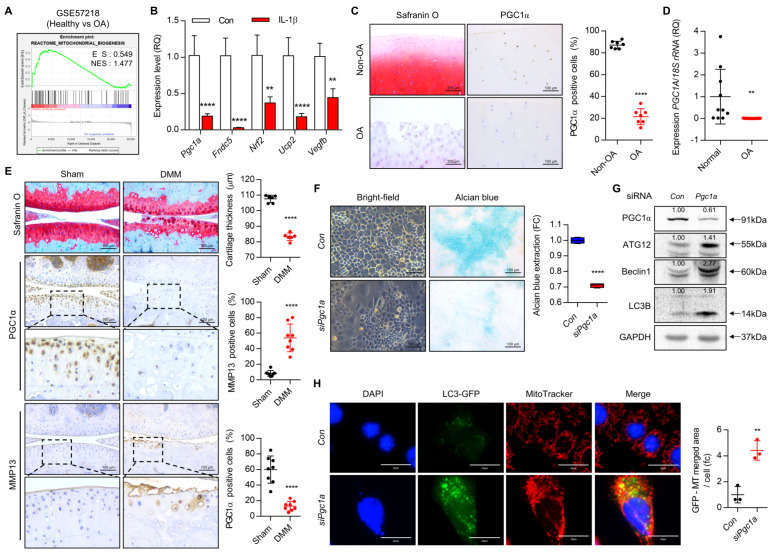
PGC1α is a key regulator for OA pathogenesis. (**A**) Gene set enrichment analysis (GSEA) of GSE57218 (seven healthy chondrocytes vs. 33 OA chondrocytes). (**B**) Transcription level of *Pgc1a* and its target genes were analyzed using qRT-PCR (*n* = 3). (**C**) Representative images of Safranin O and PGC1α staining in human Non-OA and OA cartilage, and ratio of PGC1α positive cells (*n* = 7; Scale bars, Safranin O = 200 μm, PGC1α = 100 μm). (**D**) The transcription level of *Pgc1a* in human normal and OA chondrocytes was analyzed using qRT-PCR (Normal *n* = 10; OA *n* = 17). (**E**) Representative images of Safranin O, PGC1α, and MMP13 in mouse sham and DMM cartilages (Scale bars. Cartilage thickness (*n* = 6) and positive cells for MMP13 and PGC1α (*n* = 8) were counted. (**F**) Representative images of cell morphology and alcian blue staining (*n* = 5; Scale bars, 100 μm). Alcian blue staining extracted with 6M guanidine-HCl was measured in 600 nm absorbance (*n* = 4). (**G**) Translation level of PGC1α, ATG12, Beclin1, LC3B with the introduction of *siPgc1a* into iMACs. The GAPDH antibody was used for loading control. Each protein level was measured using ImageJ software and normalized by GAPDH expression level and indicated by fold change. (**H**) Representative images of LC3-GFP puncta and MitoTracker in *Con* and *siPgc1a* transfected iMACs (*n* = 5; Scale bars, 20 μm). Results are representative of at least three independent experiments. Values were expressed as means ± s.d. An unpaired *t*-test or one-way ANOVA was used for statistical analysis. ** *p* < 0.01, **** *p* < 0.0001.

The analysis of GSE57218 (seven healthy chondrocytes vs. 33 OA chondrocytes) suggested that mitochondria biogenesis is suppressed in OA chondrocytes compared to normal chondrocytes (Figure 1A). Exposure of IL-1β into iMACs showed a significant decrease in the expression level of *Pgc1a*, a key regulator of mitochondria biogenesis [42,43] and its target genes such as *fibronectin type III domain containing 5* (*Fndc5*), *nuclear factor*, *erythroid 2 like 2* (*Nrf2*), *uncoupling protein 2* (*Ucp2*), and *vascular endothelial growth factor B* (*Vegfb*) (Figure 1B). In human cartilage, the expression level of *Pgc1a* was significantly reduced in the severely damaged area (OA) compared to a relatively healthy area (Non-OA) of OA cartilage (Figure 1C,D). OA-induced mice by destabilization of the medial meniscus (DMM) surgery, a standard for studying the onset and progression of OA [44,45] also displayed a dramatic decrease in the number of PGC1α-positive cells in cartilage compared to sham cartilage (Figure 1E). The efficiency of DMM surgery was confirmed by an increased level of MMP13, a typical cartilage matrix-degrading enzyme (Figure 1E).

To investigate the role and function of PGC1α in detail, we introduced three small interference RNA specific to *Pgc1a* (*siPgc1a*-A, -B, and -C) into iMACs and confirmed its efficiency (Supplementary Figure S3A). Among three *siPgc1a* tested, we used one siRNA (siRNA-A) that suppressed the expression level of Pgc1a most significantly. Introduction of *Pgc1a* siRNA into iMACs significantly reduced the intensity of Alcian blue staining (Figure 1F) and the number of mitotracker-positive cells and increased depolarization of mitochondrial membrane potential compared to control (Supplementary Figure S3B,C). The expression level of genes in the synthesis of cartilage matrix such as *Acan* and *Col2a1* was significantly decreased whereas the expression level of cartilage degrading enzyme such as *Mmp13* and *Adamts5* was significantly increase by knockdown of *Pgc1a* (Supplementary Figure S3D).

### 3.2. Suppression of PGC1a Activates PRKN-Independent Mitophagy through Upregulation of BNIP3

**Figure 2:**
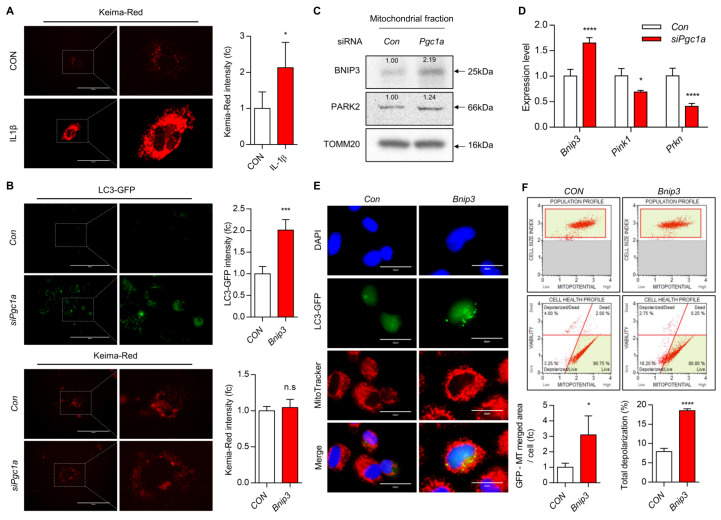
PGC1α suppression is associated with BNIP3-induced mitophagy during OA pathogenesis. (**A**) Representative images of Keima-Red with or without IL-1β in iMACs (*n* = 4; Scale bars, 100 μm). Results are representative of at least five independent experiments. (**B**) Representative images of LC3-GFP and Keima-Red with the introduction of *siPgc1a* into iMACs (*n* = 5; Scale bars, 100 μm). The results are representative of at least four independent experiments. (**C**) The mitochondrial protein expression level of BNIP3, PARK2 with the introduction of *siPgc1a* into iMACs. TOMM20 was used for loading control. Each protein level was measured using ImageJ software and normalized by TOMM20 expression level and indicated by a fold change. (**D**) The transcription level of mitophagy genes was analyzed using qRT-PCR (*n* = 3). (**E**) Representative images of LC3-GFP and MitoTracker with the introduction of *Bnip3* into iMACs (*n* = 5; Scale bars, 20 μm). Results are representative of at least five independent experiments. (**F**) Mitochondria membrane potential level was analyzed using MUSE Cell Analyzer (*n* = 3). Values were expressed as means ± s.d. An unpaired *t*-test or one-way ANOVA was used for statistical analysis. * *p* ≤ 0.05, n.s., non-significant, *** *p* < 0.001, **** *p* < 0.0001.

Recently accumulating evidence also demonstrates the pivotal role of autophagy in the pathogenesis of OA [46,47]. Autophagy is an important part of the cellular process in maintaining cartilage homeostasis. Autophagy plays a dual role in chondrocyte fates. Essentially, autophagy can play a protective role while it also can lead to chondrocyte death [48]. Our laboratory and others have suggested that the loss of key regulators in autophagic response develops OA and results in chondrocyte death [6,23,24]. Mitophagy is a special form of autophagy to maintain mitochondrial homeostasis by eliminating damaged mitochondria and unfolded proteins [49]. Impaired mitophagy is associated with mitochondrial dysfunction and apoptosis of chondrocytes in a variety of pathological processes [15,16,50]. Deregulated mitophagy accumulates defective mitochondria and leads to apoptotic cell death and ECM degradation, contributing to cartilage degeneration [50,51]. We also observed that the introduction of *siPgc1a* into iMACs significantly increased the expression level of autophagy-related proteins such as ATG12, Beclin1, and LC3B (Figure 1G). Moreover, we also observed the increased number of co-localization of MitoTracker staining and LC3-GFP puncta with *Pgc1a* knockdown in iMACs (Figure 1H), suggesting that *Pgc1a* knockdown stimulates mitophagy. Consistent with previous reports [33], under OA conditions induced by IL-1β, we observed that PINK1-dependent mitophagy was increased as visualized mitophagy with mitophagy detection vectors containing the mitochondria-targeted Keima-Red gene and the Parkin gene (Keima-Red) (Figure 2A).

Knockdown of *Pgc1a* into iMACs also increased autophagy as assessed by LC3-GFP punctata (Figure 2B). However, PINK1-dependent mitophagy was not altered by *Pgc1a* knockdown. Interestingly, the transcriptional and translational level of BNIP3 was significantly increased by *Pgc1a* knockdown (Figure 2C,D). The over-expression of *Bnip3* into iMACs was increased the number of co-localizations of MitoTracker staining and LC3-GFP puncta (Figure 2E). The depolarization of the mitochondria membrane was significantly increased (Figure 2F). Furthermore, decreased levels of *Acan* and *Col2a1* and increased levels of *Mmp13* and *Adamts5* were observed in *Bnip3*-introduced iMACs (Supplementary Figure S4).

### 3.3. miR-126-5p Is Key Regulator for PGC1a during OA Pathogenesis

**Figure 3:**
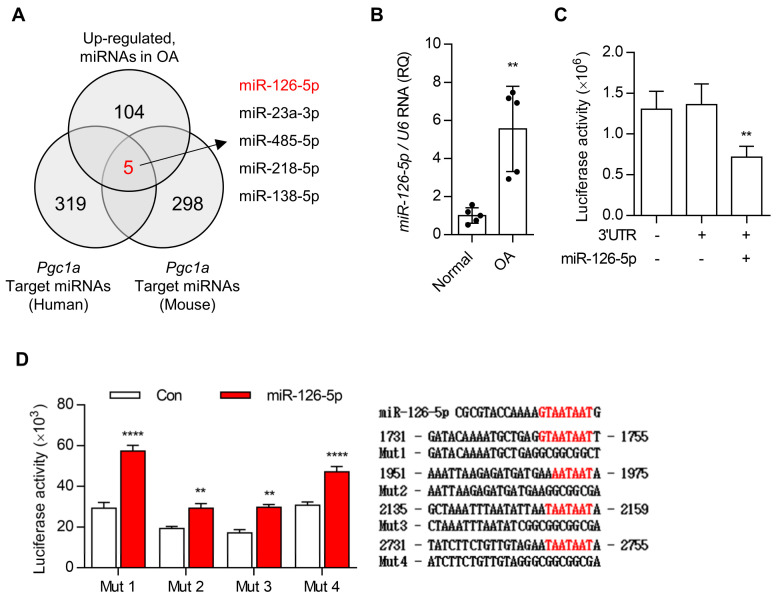
miR-126-5p regulates the expression level of BNIP3 via direct targeting. (**A**) In silico analysis using miRDB and PubMed. (**B**) The expression level of miR-126-5p in normal and OA chondrocytes were analyzed using qRT-PCR (*n* = 5). (**C**,**D**) Luciferase reporter assays of cells expressing the construct containing the *Pgc1a*-3′UTR or mutated seed sequence of targets in the absence or presence of miR-126-5p (*n* = 3). Scramble-miR was used as control (Con). Values were expressed as means ± s.d. An unpaired *t*-test or one-way ANOVA was used for statistical analysis. ** *p* < 0.01, **** *p* < 0.0001.

To search for an upstream regulator of *Pgc1a* during OA pathogenesis, we applied in silico analysis using miRDB and PubMed to extract common miRs between *Pgc1a*-targeting miR and OA-involved miR (Figure 3A). Among the 109 upregulated miRs known to involve in OA pathogenesis, 324 miRs targeting *Pgc1a* in humans, and 303 miRs targeting *Pgc1a* in mice, 5 miRs, miR-126-5p, miR-23a-3p, miR-485-5p, miR-218-5p, and miR-138-5p were common miRs and the expression level of miR-126-5p was most significantly increased both in human OA chondrocytes and IL-1β-treated iMACs (Figure 3A,B, Supplementary Figure S5A). The overexpression of miR-126-5p using its mimics suppressed the expression level of *Pgc1a* (Supplementary Figure S5B). We cloned the 3′ UTRs of *Pgc1a* into luciferase constructs. Reporter assays with miR-126-5p expressing cells independently confirmed that miR-126-5p represses *Pgc1a* (Figure 3C). The mutation of the putative miR-126-5p sites abrogated repression by miR-126-5p, thus confirming the functionality of the sites (Figure 3D).

**Figure 4:**
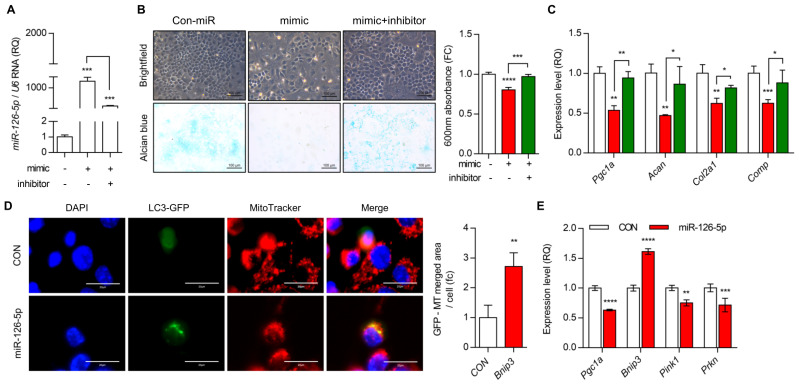
miR-126-5p dysregulates the homeostasis of cartilage matrix. (**A**) Efficiency of miR-126-5p mimic and inhibitor was confirmed by real-time PCR using iMACs. Scramble-miR was used as control (Con-miR). (**B**) iMACs seeded with the density of 1 × 10^4^/24 well culture dish were transfected with miR-126-5p mimic or inhibitor, stained with Alcian blue (left panel) and quantified based on absorbance at 600 nm (right panel). The results shown are representative of at least three independent experiments. (**C**) The transcription level of *Pgc1a*, *Acan*, *Col2a1*, and *Comp* were analyzed using qRT-PCR (*n* = 3). (**D**) Representative images of LC3-GFP and MitoTracker with the introduction of miR-126-5p mimic into iMACs (*n* = 5; Scale bars, 20 μm). Results are representative of at least five independent experiments. (**E**) Transcription level of *Pgc1a*, *Bnip3*, *Pink1*, and *Prkn* were analyzed using qRT-PCR (*n* = 3). Values were expressed as means ± s.d. An unpaired *t*-test or one-way ANOVA was used for statistical analysis. * *p* ≤ 0.05, ** *p* < 0.01, *** *p* < 0.001, **** *p* < 0.0001.

To verify the role of miR-126-5p in maintaining cartilage homeostasis, cells were treated with miR-126-5p mimics. The cartilage matrix was assessed by alcian blue staining (Figure 4A,B). The expression level of genes in the cartilage matrix synthesis, i.e., *Acan*, *Col2a1*, and *Comp* were significantly decreased, whereas the expression level of genes in degradation of cartilage matrix, i.e., *Mmp3*, *Mmp9*, *Mmp13*, *Adamts4*, and *Adamts5* was significantly increased by the overexpression of miR-126-5p (Figure 4C and Supplementary Figure S5C). Moreover, the exposure of miR-126-5p mimics into iMACs increased the number of co-localizations of MitoTracker staining and LC3-GFP puncta (Figure 4D).

**Figure 5:**
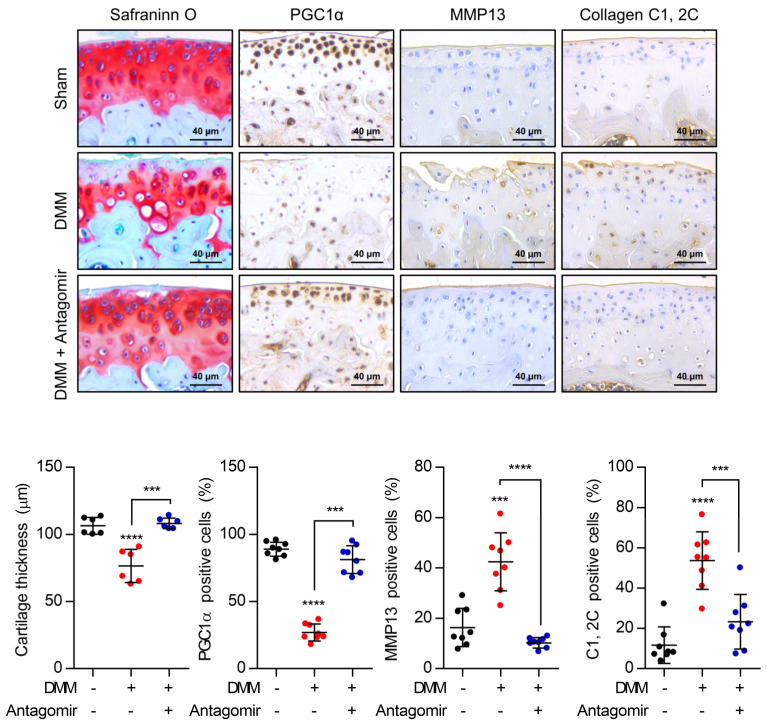
miR-126-5p dysregulates the homeostasis of the cartilage matrix. Mouse cartilages induced by destabilization of the medial meniscus (DMM) were infected with lentiviruses containing miR-126-5p inhibitor (Antagomir). The expression level of PGC1a, MMP13, and Collagen C1, 2C were analyzed (upper panel). Positive cells were counted as three different fields/experiments, averaged, and represented as a dot graph (lower panel). Results are representative of at least three independent experiments. Values were expressed as means ± s.d. An unpaired *t*-test or one-way ANOVA was used for statistical analysis. *** *p* < 0.001, **** *p* < 0.0001.

The expression level of Bnip3 was increased with exposure of miR-126-5p mimics, not the expression level of two factors in PINK1-dependent mitophagy, Pink1 and PAKIN (Figure 4E). The exposure of miR-126-5p inhibitor into iMACs in the presence of miR-126-5p mimics recovered the expression level of genes in cartilage matrix synthesis and degradation modulated by treatment of miR-126-5p mimics (Figure 4C). Moreover, in a DMM-induced OA mouse, cartilage degradation was significantly inhibited by the introduction of miR-126-5p inhibitor (Figure 5). The introduction of miR-126-5p inhibitor also increased the number of PGC1α-positive cells and decreased the number of MMP13, C1,2C-positive cells in DMM-induced OA cartilage.

## 4. Discussion

Mitochondria play a role in regulating and modulating the redox state and various biochemical reactions to maintain the internal cellular signaling such as AMPK or calcium signaling in normal chondrocytes. Chondrocytes produce ATP mainly by glycolysis but 25% of total ATP production occurs through mitochondrial oxidative phosphorylation [51]. It has been suggested that decreased mitochondrial activity and increased depolarization of the mitochondria membrane in OA chondrocytes stimulate an inflammatory response [8,52].

Impaired mitochondrial biogenesis is known in OA chondrocytes. Mitochondrial dysfunction is linked to OA characteristics such as decreasing synthesis of cartilage matrix and the upregulation of matrix metalloproteinase and results in cartilage degradation. In mitophagy, a mechanism of intracellular catabolism, damaged mitochondria is removed and plays an essential role in maintaining mitochondrial quality control and homeostasis [53]. Until now, several mitochondrial receptors, such as BNIP3, NIX/BNIP3L, FUNDC1 and regulatory proteins such as autophagy and Beclin 1 regulator 1 (AMBRA), mitochondrial E3 ubiquitin protein ligase 1 (MUL1), autocrine motility factor receptor (AMFR), SMAD specific E3 ubiquitin protein ligase 1 (SMURF1), and ras homolog, mTORC1 binding (RHEB) in the molecular mechanism of mitophagy has been reported [30,54,55].

Defective mitophagy is associated with various diseases including neurodegenerative diseases, cancer, and metabolic diseases, suggesting a close link between human disease and mitophagy [56,57]. Different pathways in regulating mitophagy are known, and the best-studied pathway is mediated by the phosphatase and tensin homologue (PTEN)-induced putative kinase 1 (PINK1) and the Parkin, an E3 ubiquitin ligase in mitochondrial outer membrane [58]. PRKN-dependent mitophagy is also relatively well-studied in OA pathogenesis. Impaired mitophagy by PRKN in OA chondrocytes increased mitochondrial dysfunction and ROS production whereas active parkin eliminates damaged mitochondria and prevent the induction of oxidative stress [50].

Peroxisome proliferator-activated receptor γ coactivator 1α (PGC-1α), a known master regulator of mitochondrial biogenesis, regulates energy metabolism and mitochondria homeostasis [42]. Reduction of PGC-1α expression has been reported in mouse knee cartilage with OA and aging [18,59]. In chondrocytes, PGC-1α is known to inhibit catabolic responses via the activation of AMPK and the activation of AMPK-SIRT1- PGC-1α pathway in OA chondrocytes reversed impaired mitochondrial capacity [18]. Here, we also found the reduced level of PGC-1α expression in human OA cartilage.

PGC-1α is known to improve the mitochondria function by stimulating antioxidant capability [60], by regulating the expression level of genes in mitochondrial fusion, such as mitofusion 1 and 2 [61]. PGC1α has also been reported to stimulate the autophagy factors such as LC3B and p62 in skeletal muscle [62] and the co-expression of PGC1α and Parkin is known to increase the mitochondria number and accelerate the recovery of the mitochondrial membrane potential in cortical neurons [63]. Recently, it has been suggested that the over-expression of PGC1α inhibited FoxO3-mediated transcriptional activity [64,65] that drives the expression of multiple mitophagy factors such as Mul1 and Bnip3 [66]. Here, we found that a lack of PGC1α results in the activation of PRKN-independent mitophagy through the upregulation of Bnip3 during OA pathogenesis. However, the precise molecular mechanisms of how Bnip3 mediated mitophagy remain unclear. It has been suggested that Bnip3 could induce mitophagy via promoting a mitochondrial depolarization or serve directly as mitophagy receptors through the binding of an LC3-interacting region (LIR) to LC3B on autophagosomes [67,68].

Our study demonstrated that the overexpression of Bnip3 into iMACs induced the defects in mitochondrial depolarization, suggesting that this promoted mitochondrial depolarization by Bnip3 could sufficiently induce mitophagy and result in the imbalance of cartilage matrix homeostasis. Since the upregulated expression of Bnip3 has been reported in OA cartilage of OA patients, and suggested the positive correlation between Bnip3 and OA chondrocyte apoptosis [69], the regulation of Bnip3-induced mitophagy suggested in this paper could be a potent therapeutic strategy for controlling OA pathogenesis. However, the precise and detailed regulatory mechanisms of mitochondrial PGC-1α: Bnip3 interaction during OA pathogenesis need to be further studied.

In sum, our study suggests that miR-126-5p targeted 3′UTR of PGC-1α and suppressed the expression level of PGC1α. A reduced level of PGC1α expression in OA chondrocytes activated the PRKN-independent mitophagy through the upregulation of Bnip3 and stimulated cartilage degradation and the apoptotic death of chondrocytes. Therefore, the identification of pharmacological targets along the PGC1α: BNIP3 mitophagy axis could be of therapeutic benefit to cartilage-degrading diseases.

## 5. Conclusions

We suggested that a reduced level of PGC1α due to an increased level of miR-126-5p activated PRKN-independent mitophagy through the upregulation of Bnip3 and stimulated cartilage degradation and apoptotic death of chondrocytes during OA pathogenesis.

## Data Availability

The data presented in this study are available in the manuscript.

## Supplementary Materials

The following are available online at https://www.mdpi.com/article/10.3390/cells10071839/s1, Figure S1: Mitochondria dysfunction during OA pathogenesis; Figure S2: Mitochondria dysfunction dysregulates the homeostasis of cartilage matrix; Figure S3: Mitochondria dysfunction dysregulates the homeostasis of cartilage matrix; Figure S4: Transcription level of *Bnip3*, *Acan*, *Col2a1*, *Mmp13* and *Adamts5* with introduction of *Bnip3* into iMACs were analyzed using qRT-PCR; Figure S5: The involvement of miR-126-5p in the pathogenesis of OA. Table S1: The list of primers used.
