# Supplementary material for: BNIP3-Dependent Mitophagy via PGC1α Promotes Cartilage Degradation
Source: Cells. 2021 Jul 20;10(7):1839. doi: 10.3390/cells10071839 (PMC8304751; doi:10.3390/cells10071839)
Supplement: Supplementary file 1 [file cells-10-01839-s001.zip › cells-1246485-supplementary.pdf]

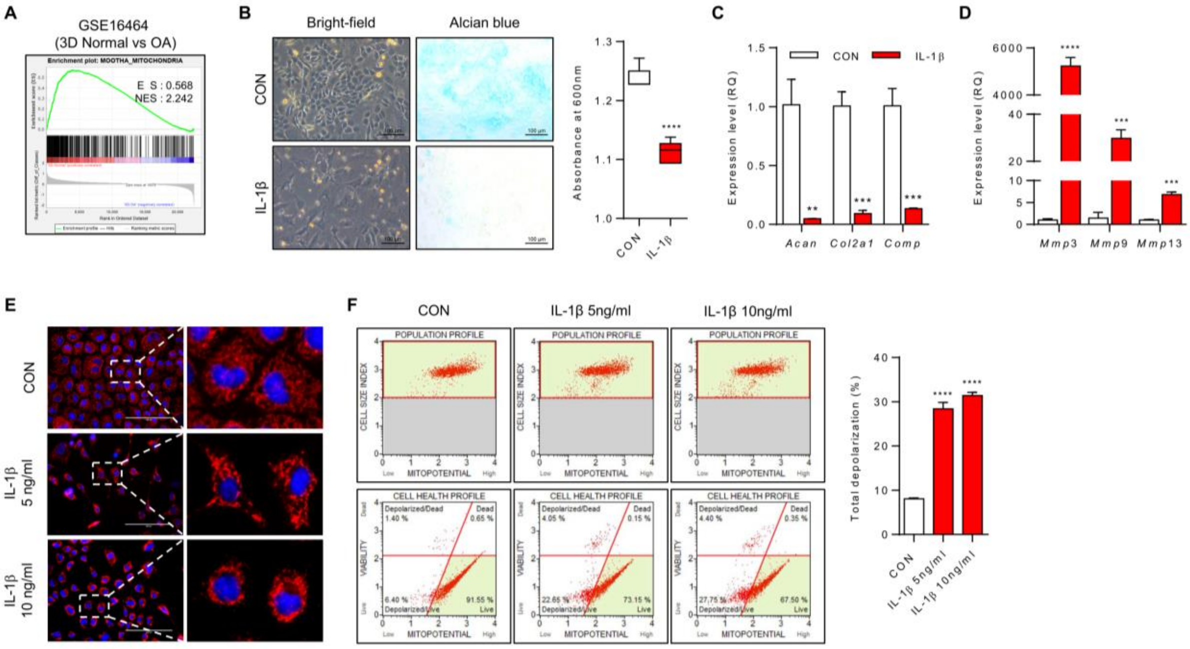

Supplementary Figure S1. Mitochondria dysfunction during OA pathogenesis. **(A)** Gene set enrichment analysis (GSEA) of GSE16464 (3D culture of normal vs. OA chondrocyte). **(B)** iMACs were treated with IL-1β and stained with Alcian blue. Alcian blue staining extracted with 6M guanidine-HCl was measured in 600nm absorbance (n = 4). **(C)** Transcription level of *Acan*, *Col2a1*, and *Comp* were analyzed using qRT-PCR (n = 3). **(D)** Transcription level of of *Mmp3*, *Mmp9*, and *Mmp13* were analyzed using qRT-PCR (n = 3). **(E)** Representative images of MitoTracker with IL-1β-treated iMACs (n = 5; Scale bars, 20μm). **(F)** Mitochondria membrane potential level was analyzed using MUSE Cell Analyzer (n = 3). Values were expressed as means + s.d. An unpaired *t*-test or one-way ANOVA were used for statistical analysis. ***P < 0.001, ****P < 0.0001.

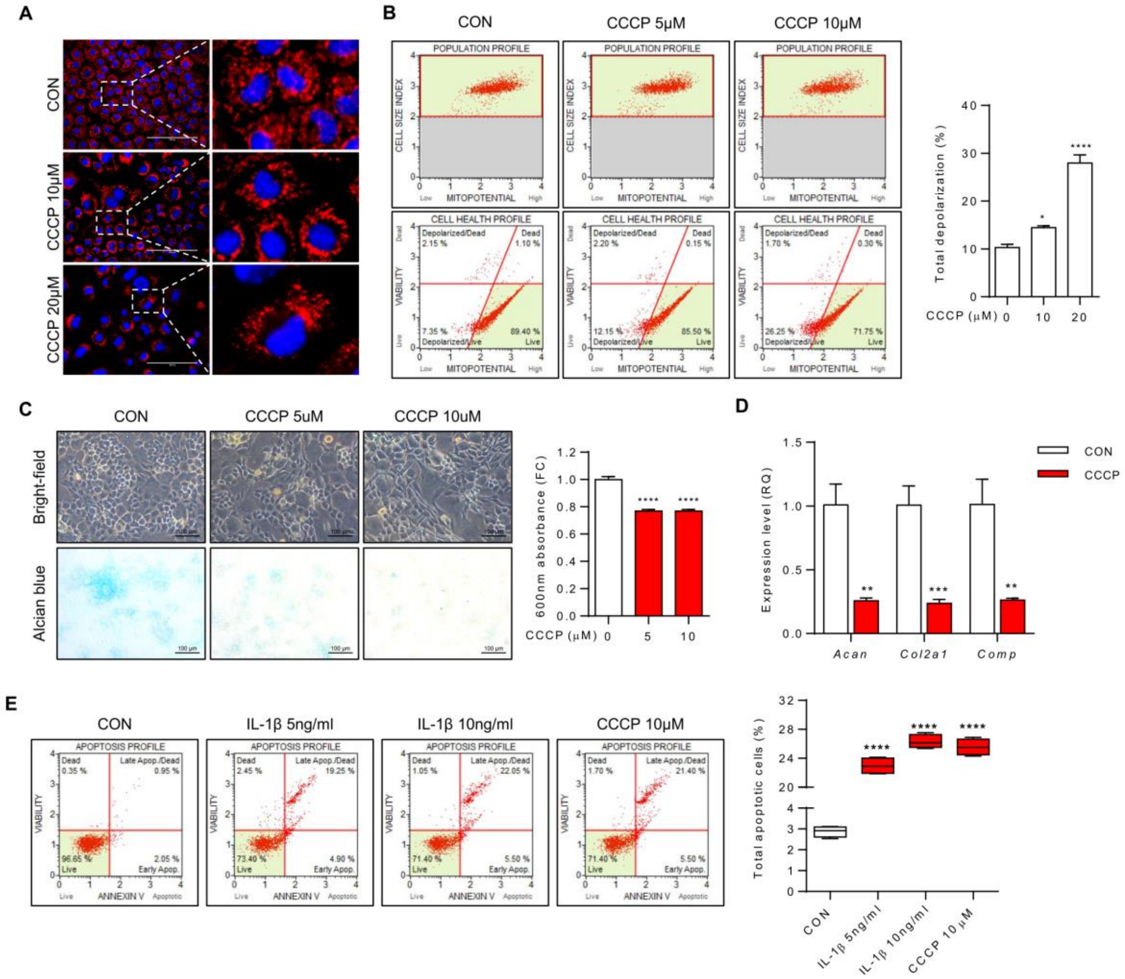

Supplementary Figure S2. Mitochondria dysfunction dysregulates the homeostasis of cartilage matrix. **(A)** iMACs were treated with carbonyl cyanide 3-chlorophenylhydrazone (CCCP) and stained with MitoTracker (n = 5; Scale bars, 20μm). **(B)** Mitochondria membrane potential level was analyzed using MUSE Cell Analyzer (n = 3). **(C)** Representative images of Alcian blue with CCCP-treated iMACs (Scale bars, 100μm). Alcian blue staining extracted with 6M guanidine-HCl was measured in 600nm absorbance (n = 4). **(D)** Transcription level of *Acan*, *Col2a1*, and *Comp* were analyzed using qRT-PCR (n = 3). **(E)** Apoptotic cell death was analyzed using MUSE Cell Analyzer (n = 3). Values were expressed as means + s.d. An unpaired *t*-test or one-way ANOVA were used for statistical analysis. *P ≤ 0.05, ***P < 0.001, ****P < 0.0001.

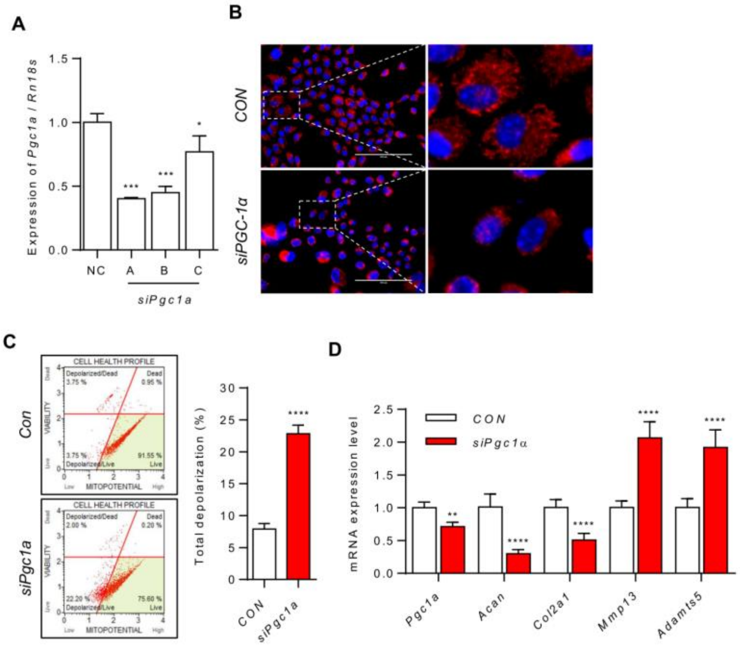

Supplementary Figure S3. Mitochondria dysfunction dysregulates the homeostasis of cartilage matrix. **(A)** Efficiency of three different *siPgc1a* (A, B, C) was confirmed by real-time PCR using iMACs. **(B)** Representative images of MitoTracker with introduction of *siPgc1a* into iMACs (n = 5; Scheme 100. m). **(C)** Mitochondria membrane potential level was analyzed using MUSE Cell Analyzer (n = 3). **(D)** Transcription level of *Pgc1a, Acan*, *Col2a1*, *Mmp13* and *Adamts5* were analyzed using qRT-PCR (n = 3). Values were expressed as means + s.d. An unpaired *t*-test or one-way ANOVA were used for statistical analysis. *P ≤ 0.05, ***P < 0.001, ****P < 0.0001.

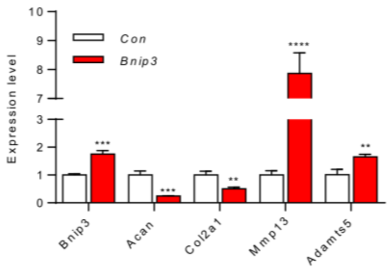

Supplementary Figure S4. Transcription level of *Bnip3, Acan*, *Col2a1*, *Mmp13* and *Adamts5* with introduction of *Bnip3* into iMACs were analyzed using qRT-PCR (n = 3).

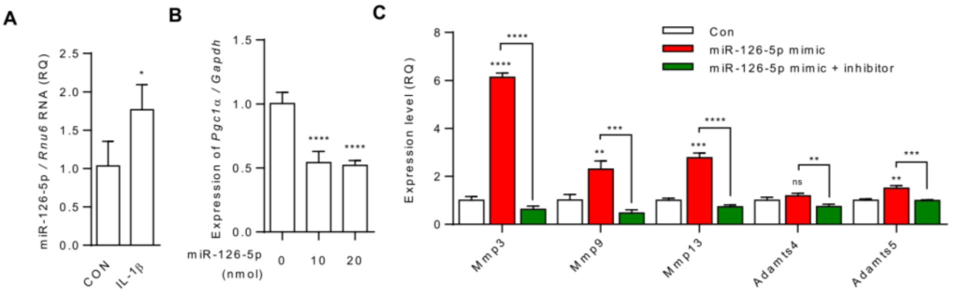

Supplementary Figure S5. The involvement of miR-126-5p in the pathogenesis of OA. **(A)** The expression level of miR-126-5p in iMACs treated with IL-1β. **(B)** The expression level of *Pgc1a* in iMACs treated with miR-126-5p. **(C)** Transcription level of *Mmp3*, *Mmp9*, *Mmp13*, *Adamts4*, and *Adamts5* in iMACs treated with miR-126-5p mimic or miR-126-5p inhibitor were analyzed using qRT-PCR (n = 3). Values were expressed as means + s.d. An unpaired *t*-test or one-way ANOVA were used for statistical analysis. *$P \leq 0.05$, ***$P < 0.001$, ****$P < 0.0001$.

Supple.

#Western blot uncropped image

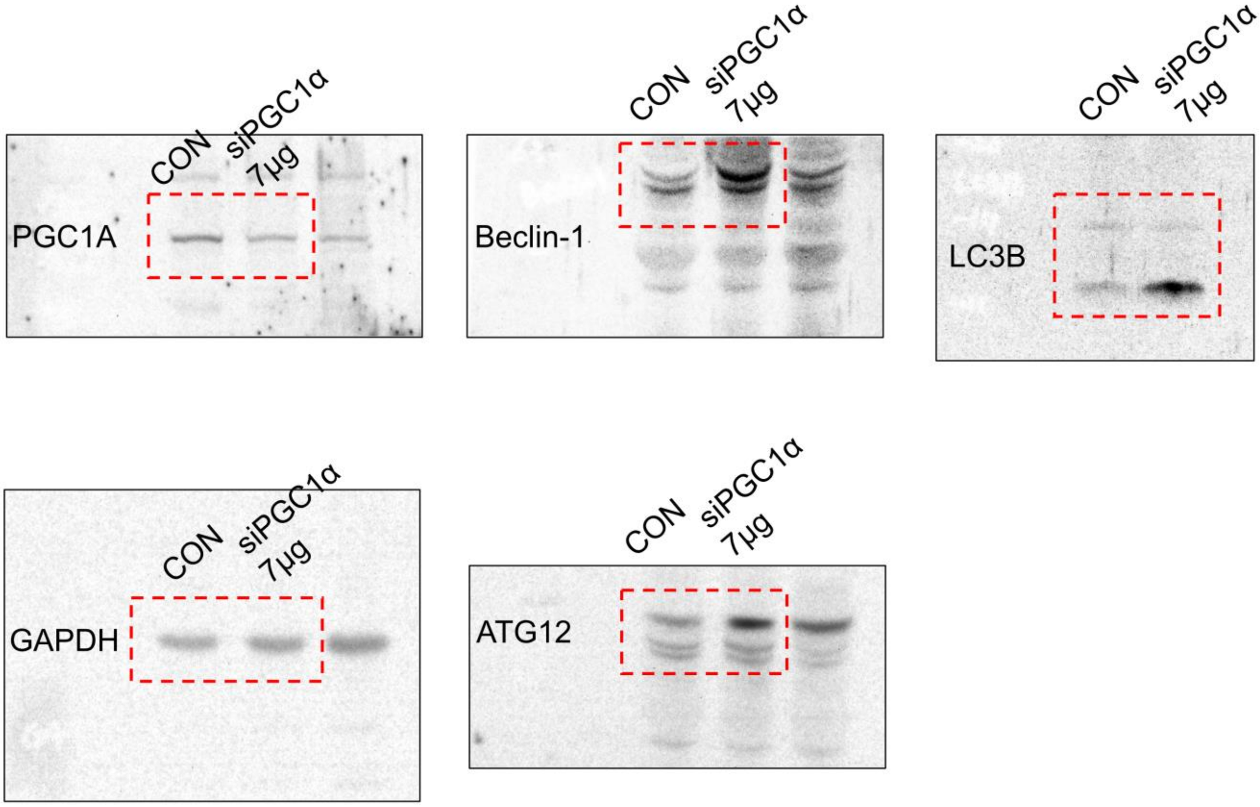

#Western blot uncropped image

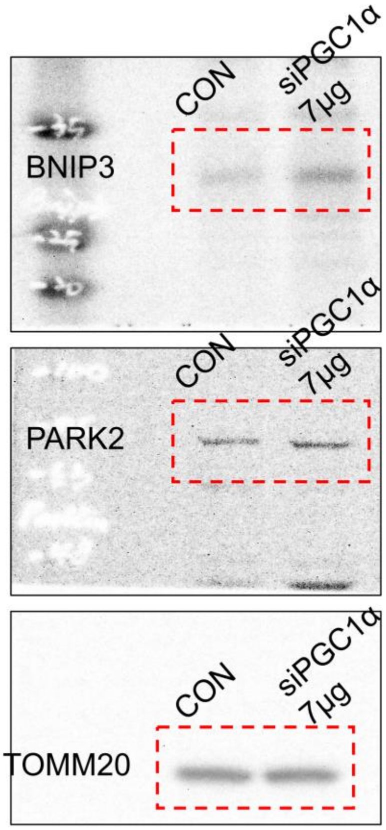

Supplementary Table S1. The primers used in this study

**Gene name**	**Forward (5′ → 3′)**	**Reverse (5′ → 3′)**
Human *PGC1A*	TGAACTGAGGGACAGTGATTTC	CCCAAGGGTAGCTCAGTTTATC
Human *RN18S*	CTGAGAAACGGCTACCACATC	GCCTCGAAAGAGTCCTGTATTG
Mouse *Pgc1a*	CTAGCCATGGATGGCCTATTT	GTCTCGACACGGAGAGTTAAAG
Mouse *Fndc5*	GAGGTGACCATGAAGGAGATG	GCGGCAGAAGAGAGCTATAA
Mouse *Nrf2*	CTCCGTGGAGTCTTCCATTTAC	GCACTATCTAGCTCCTCCATTTC
Mouse *Ucp2*	CCAGCCTACAGATGTGGTAAAG	TCGACAGTGCTCTGGTATCT
Mouse *Vegfb*	CATGGGCAATGTGGTCAAAC	AGGATCTGCATTCGGACTTG
Mouse *Bnip3*	TCCAGCCTCCGTCTCTATTT	CTGTCACAGTGAGAACTCTTGG
Mouse *Pink1*	GTGGAATATCTCGGCAGGTT	CTCCATACTCTCCAGCCAAAG
Mouse *Prkn*	CCTTCTGCCGGGAATGTAAA	GGCTCTTTCATCGACTCTGTAG
Mouse *Acan*	GAGACTTCTGCCTCTGGAATAG	CTCCAGAAGGAATCCCACTAAC
Mouse *Col2a1*	CTGGTTTGGAGAGACCATGAA	GAGGAAAGTCATCTGGACGTTAG
Mouse *Comp*	CGTGGGCTGGAAGGATAAA	TACTAGCTCAGGACCCTCATAG
Mouse *Mmp3*	GGACCAGGGATTAATGGAGATG	TGAGCAGCAACCAGGAATAG
Mouse *Mmp9*	TCTGTATGGTCGTGGCTCTAA	GGAGGTATAGTGGGACACATAGT
Mouse *Mmp13*	CCCTGATGTTTCCCATCTATACC	TTCATCGCCTGGACCATAAAG
Mouse *Adamts5*	TGCCACAGACCCAACTAAAG	CCATGGCTGATGACAGAGTT
Mouse *Rn18s*	CTGAGAAACGGCTACCACATC	GCCTCGAAAGAGTCCTGTATTG
